# Even low levels of tree cover improve dietary quality in West Africa

**DOI:** 10.1093/pnasnexus/pgae067

**Published:** 2024-02-09

**Authors:** Bowy den Braber, Charlotte Hall, Martin Brandt, Florian Reiner, Maurice Mugabowindekwe, Laura Vang Rasmussen

**Affiliations:** Department of Geosciences and Natural Resource Management, University of Copenhagen, Øster Voldgade 10, 1350 Copenhagen K, Denmark; Department of Geosciences and Natural Resource Management, University of Copenhagen, Øster Voldgade 10, 1350 Copenhagen K, Denmark; Department of Biological and Environmental Sciences, University of Stirling, Stirling FK9 4LA, UK; Department of Geosciences and Natural Resource Management, University of Copenhagen, Øster Voldgade 10, 1350 Copenhagen K, Denmark; Department of Geosciences and Natural Resource Management, University of Copenhagen, Øster Voldgade 10, 1350 Copenhagen K, Denmark; Department of Geosciences and Natural Resource Management, University of Copenhagen, Øster Voldgade 10, 1350 Copenhagen K, Denmark; Department of Geosciences and Natural Resource Management, University of Copenhagen, Øster Voldgade 10, 1350 Copenhagen K, Denmark

## Abstract

Forests are attracting attention as a promising avenue to provide nutritious and “free” food without damaging the environment. Yet, we lack knowledge on the extent to which this holds in areas with sparse tree cover, such as in West Africa. This is largely due to the fact that existing methods are poorly designed to quantify tree cover in drylands. In this study, we estimate how various levels of tree cover across West Africa affect children's (aged 12–59 months) consumption of vitamin A–rich foods. We do so by combining detailed tree cover estimates based on PlanetScope imagery (3 m resolution) with Demographic Health Survey data from >15,000 households. We find that the probability of consuming vitamin A–rich foods increases from 0.45 to 0.53 with an increase in tree cover from the median value of 8.8 to 16.8% (which is the tree cover level at which the predicted probability of consuming vitamin A–rich foods is the highest). Moreover, we observe that the effects of tree cover vary across poverty levels and ecoregions. The poor are more likely than the non-poor to consume vitamin A–rich foods at low levels of tree cover in the lowland forest-savanna ecoregions, whereas the difference between poor and non-poor is less pronounced in the Sahel-Sudan. These results highlight the importance of trees and forests in sustainable food system transformation, even in areas with sparse tree cover.

**Significance Statement**

Better quality diets are key to solving the widespread problem of nutrient deficiencies. Yet, far too often, food and nutrition security policies focus on increasing agricultural production and access to sufficient calories as the main solution, which often results in degraded ecosystems. In this study, we examine the empirical relationship between having forests and trees in the surroundings and the probability of children consuming nutritious foods. Based on a sample consisting of >15,000 rural households living in West Africa, we show that even low levels of tree cover improve the likelihood of children aged 12–59 months consuming vitamin A–rich foods.

## Introduction

The current global food system is detrimental to both human and environmental health, with 800 million people undernourished and around 2 billion consuming low-quality diets that can lead to micronutrient deficiencies, and dramatically rising rates of noncommunicable diseases, such as diabetes (1, 2). At the same time, agriculture is responsible for about a third of global greenhouse gas emissions (3), is the leading driver of deforestation (4), is the largest user of global freshwater withdrawals, and is a major source of both freshwater and ocean pollution (5). A growing body of literature has shown the potential of forests and trees for providing dual benefits for both environmental sustainability and human health, primarily in middle- and low-income countries. For example, living in close proximity to forests has been shown to increase dietary diversity and consumption of nutrient-dense foods (6–10). Likewise, rural smallholders who incorporate trees on or around their farms have improved food security outcomes (11). However, there is a dearth of empirical evidence on whether these relationships hold in areas with sparse tree cover, despite such areas covering large parts of the world (12) and providing ecosystem services to large populations (13). Although multiple case studies from West Africa have indicated that even sparse tree cover could be positive for people's diets (14–18), methods to assess global tree cover (19) have, until now, been insufficient to quantify tree cover in drylands (12, 20), thus hindering large-scale assessments on tree-diet linkages. The recent development of methods in combination with access to imagery with a high enough resolution to identify single trees (12, 20, 21) provides an avenue to assess the effects of both forests and trees outside of forests on dietary quality. This is especially important in areas such as West Africa, our study area, because (i) there is a surprisingly high occurrence of trees outside of forests (20), albeit with their provision of a myriad of ecosystem services largely uncovered (22, 23), and (ii) rates of undernourishment and malnutrition are generally high in this region (2).

In this study, we examine the linkages between various levels of tree cover across West Africa and people's consumption of vitamin A–rich foods. We focus specifically on vitamin A–rich foods given the high rates of vitamin A deficiency across West Africa (24). Vitamin A deficiency can lead to a number of health issues, including permanent blindness, or, in less serious cases, “night blindness”. West African countries (particularly Niger, Mali, and Guinea) had the highest rates of night blindness in pregnant women in 2005 (24) (unfortunately, there are no more recent statistics), with the exception of Ethiopia, which had the highest rate worldwide. We investigate whether the effects of tree cover on people's food consumption vary across ecoregions (Sahel-Sudan and lowland forest-savanna; Fig. S1) and people's poverty levels (measured as the living standards dimension of the multidimensional poverty index [MPI] (25)). We note that our data only comes from one single time point and that there is no experimental manipulation in our study design. As such, the effects of tree cover refer to associations in real-world settings, and we make no claims about causality. To examine the effects of tree cover on people's food consumption, we combine tree cover estimates from PlanetScope imagery (3 m resolution) with Demographic Health Survey (DHS) data from >80,000 households, of which >15,000 households have children aged 12–59 months, covering 10 countries in West Africa. Our approach uses a tree cover dataset based on state-of-the-art imagery (12), which allows us to also include areas with sparse tree cover, such as the Sahel. Because rainfall is an important determinant of tree cover in the region (26), we divide our study area into two regions with different rainfall patterns using ecoregions from Olson et al. (27). The Sahel-Sudan ecoregion in the north receives much less rainfall than the lowland forest-savanna ecoregion in the south and is characterized by different tree species (28). For both regions, we examine the linkages between tree cover and people's dietary quality across different poverty levels.

The underlying assumption for our analysis rests on recent research establishing that forests can provide considerable dietary benefits to households. That is, forests can improve people's diets along four key pathways (6, 29, 30). The first and most direct pathway is through the provision of wild foods, which include foods such as mangoes (*Mangifera indica*) and dark green leafy vegetables (e.g. baobab leaves [*Adansonia digitata*]) which tend to be very rich in vitamin A, and animal products (i.e. bushmeat and insects), which are a good source of bioavailable zinc and iron. Wild foods rarely make up the majority of the diet, instead supplementing what is available from agricultural production or markets. Wild foods thus tend to increase overall dietary diversity and can improve the micronutrient adequacy of the diet (31, 32). The second pathway is through income gains from sales of nontimber forest products (NTFPs), which can facilitate the purchase of nutritious foods from markets (33). The third pathway is through the flow of ecosystem services from forests into surrounding agricultural landscapes, which can in turn increase and/or diversify agricultural production (34). The fourth pathway is through the provision of fuelwood for cooking, which can improve nutrition by facilitating the preparation of a range of foods, particularly those with long cooking times (35), such as dark green leafy vegetables, often used in relish in West Africa. However, knowledge on the relative contribution of each of these four pathways remains limited, as existing studies have typically assessed these pathways in isolation and in areas with dense tree cover (6, 30). Due to the nature of the DHS data on which we rely, it is not possible to assess the contribution of each of the four pathways. Yet, we advance existing knowledge by empirically assessing whether there is an association between tree cover and people's consumption of nutritious foods in areas with sparse tree cover. Such assessment is an important first step to uncover whether regions, such as the Sahel, with mostly sparse tree cover, exhibit the productivity and species diversity needed to improve diets.

We first examined the relationship between tree cover (in a 5-km radius around households) and the consumption of vitamin A–rich foods. The consumption of vitamin A–rich foods was a binary variable estimated using DHS data, whereby women were asked whether or not their children aged 12–59 months consumed vitamin A–rich foods within the last 24 h. We used a rigorous quasi-experimental matching technique (covariate balancing generalized propensity scores; CBGPS (36)) to adjust for the nonrandom distribution (selection bias) of tree cover, our treatment variable of interest. By controlling for key covariates likely to affect people's diets (Table S1), we were able to isolate the relationship between tree cover and the consumption of vitamin A–rich foods. The key advantage of the CBGPS approach is that it is applicable to continuous treatment variables, such as forest cover. We then combined this with generalized additive models (GAMs) (37), which have the advantage that they allow for nonlinear relationships. By combining these two approaches, our analysis presents an important step forward in teasing apart how various levels of tree cover affect consumption of a highly nutritious food group in nonlinear ways.

## Results

### Any increase in tree cover improves children's dietary quality

**Fig. 1.**
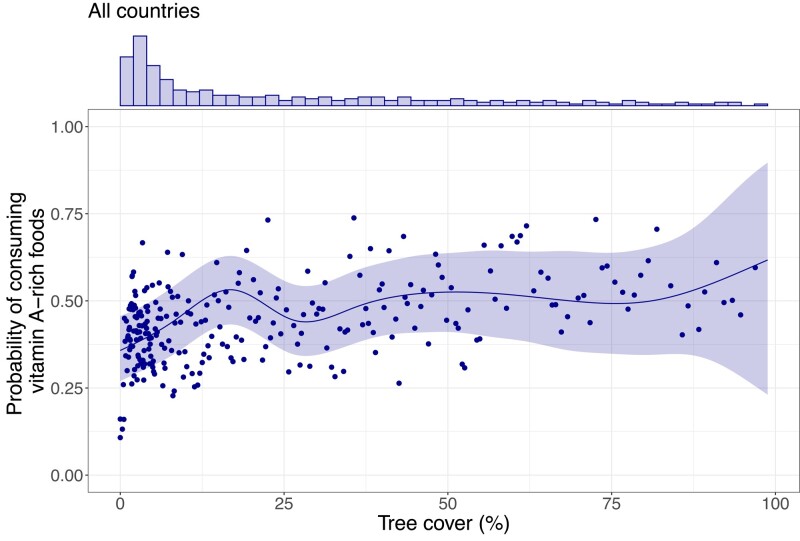
Effects of tree cover on consumption of vitamin A–rich foods. *N* = 15,875 rural households with children aged 12–59 months (81,296 rural households in total) in Senegal, Mali, Nigeria, Gambia, Benin, Guinea, Chad, Liberia, Sierra Leone, and Cameroon. The line indicates the fitted probability that the children in a household consumed vitamin A–rich foods. The shaded area shows the credible interval. The points indicate the average probability of consumption by clusters of households. The top chart shows the frequency of household observations across tree cover values, ranging from 39 to 4,579.

We found that higher levels of tree cover were associated with an increased probability of consuming vitamin A–rich foods (*P* < 0.001), especially in areas with very low levels of tree cover, where existing global assessments (19) have failed to accurately quantify tree cover (12). Specifically, our analysis revealed that an increase in tree cover from 8.8% (which is the median value across the >15,000 households with children aged 12–59 months) to 16.8% (the tree cover level at which the predicted probability of consuming vitamin A–rich foods is highest) is associated with an increase in the probability of consuming vitamin A–rich foods from 0.45 (credible interval 0.35–0.55) to 0.53 (credible interval 0.43–0.63; Fig. 1). This is a substantial increase given that just 41 and 50% of households consumed vitamin A–rich foods in the Sahel-Sudan and lowland forest-savanna ecoregions, respectively (Table S1). Moreover, we observed that when tree cover is above 30%, which is already classified as a forest (38), the effect of trees flattens. This suggests that in West Africa, trees outside of forests (i.e. low levels of tree cover that do not constitute a forest) play a more important role than trees inside forests for people's consumption of vitamin A–rich foods, which is also where most of the people reside. Our findings further advance existing studies showing that living near, and having access to, forest landscapes is beneficial for the intake of vitamin A–rich foods (17, 31, 39). Yet, our analysis shows that the effect is not linear, with the main effect occurring at sparse tree cover across West Africa.

### The effects of tree cover vary across poverty levels and ecoregions

**Fig. 2.**
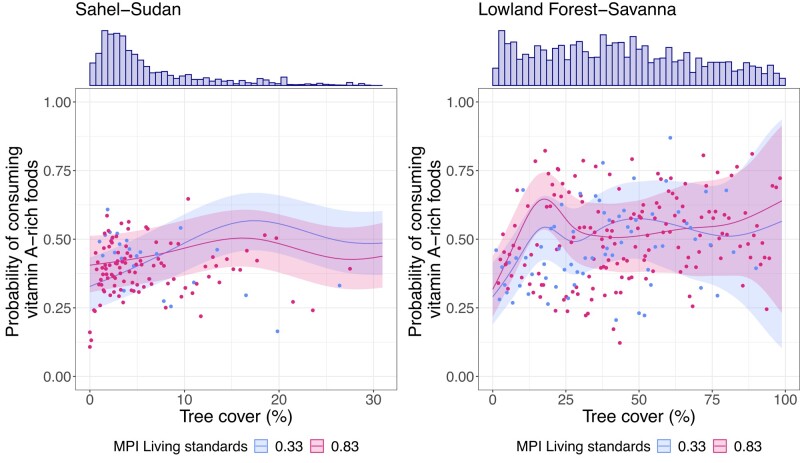
Effects of tree cover on consumption of vitamin A–rich foods per ecoregion and across poverty levels. *N* = 15,875 rural households with children under 5 years old (81,296 rural households in total) in Senegal, Mali, Nigeria, Gambia, Benin, Guinea, Chad, Liberia, Sierra Leone, and Cameroon. Lines indicate the fitted probability that the children in a household consumed vitamin A–rich foods—red line indicates a high level of poverty (MPI > 0.83—highest 20% of the population), blue line indicates a low level of poverty (MPI < 0.33—lowest 20% of the population). The shaded area shows the credible interval. The points indicate the average probability of consumption of groups of households with similar tree cover levels and MPI. The top charts show the frequency of household observations across tree cover values, ranging from 1 to 545 for the Sahel-Sudan ecoregions (*N* = 5,965), and from 11 to 136 for the Lowland Forest-Savanna ecoregions (*N* = 9,910).

We also examined whether the relationship between tree cover and children's consumption of vitamin A–rich foods differed across two separate ecoregions (27) (the Sahel-Sudan ecoregion and the lowland forest-savanna ecoregion) and poverty levels. Overall, we see that in the Sahel-Sudan ecoregion, effects of tree cover are mostly equal among the poor and non-poor households, yet the non-poor benefit marginally more at 15–25% tree cover (*P* = 0.075, Fig. 2). Potential explanations for the non-poor benefiting more may include income gains from sales of NTFPs, which can facilitate the purchase of vitamin A–rich foods from markets (33) (the second pathway potentially connecting tree cover to people's diets). In contrast, in the lowland forest-savanna ecoregion, the poor are more likely to consume vitamin A–rich foods at low levels of tree cover (*P* < 0.001). While this observation is well aligned with research showing that the poorest people often rely on the forest for collecting and consuming wild foods (40) (the first pathway potentially connecting tree cover to people's diets), it adds to this literature by illustrating that it only happens at low levels of tree cover. That is, after the consumption of vitamin A–rich foods peaks at ∼18% tree cover, we observe a drop after which consumption levels remain relatively constant and the difference between poor and non-poor disappears. The fact that the non-poor households appear to be benefiting the most from sparse tree cover in the Sahel-Sudan ecoregion, whereas the opposite pattern is seen in the lowland forest-savanna ecoregion, can be explained by different characteristics of sparse tree cover. That is, in the lowland forest-savanna ecoregions, low tree cover levels likely represent deforested areas that host semi-agricultural species such as vitamin A–rich dark green leafy vegetables (41) that can be collected for free (by the poor). Such species are generally less common in areas with naturally low tree cover, such as the Sahel-Sudan ecoregion. Low levels of tree cover in the lowland forest-savanna ecoregion could also represent agroforestry systems that host trees yielding vitamin A–rich fruits such as mango.

## Discussion

Our analysis reveals the importance of trees in areas with sparse tree cover, yet these are rarely taken into account in existing forest-diet research, primarily due to limitations in remote sensing methods that are often not designed to quantify tree cover in drylands (12). While we recognize the potential dietary benefits of trees outside of forests in landscapes with relatively sparse tree cover, it is also critical to acknowledge that existing large-scale environmental policies in Africa, such as the AFR100 and the Great Green Wall restoration initiatives, are biased toward financing reforestation initiatives focused on the planting of highly visible, fast growing tree species (42) instead of food-trees. Future initiatives could be better targeted to preserve and increase species with nutritional benefits (43). In the Sahel-Sudan ecoregions, examples include *Moringa oleifera* and *A. digitata* (baobabs)—both species that are able to grow in areas with sparse tree cover. Other examples of commonly observed species include *Annona senegalensis* (wild custard apple) and *Salvadora persica* (the toothbrush tree) (Table S2). Trees in the Sahel-Sudan might be especially important for other purposes too because they also serve as an important source of fodder, fuelwood, and fiber, and provide regulating and supporting ecosystem services (22). In lowland forest-savanna areas with sparse tree cover, reforestation initiatives will have to account for the fact that these areas have likely already been deforested and any species of value will have been removed. Instead, such areas are likely inhabited by early-successional pioneer species, and invasive and exotic species (many of which are edible). As such, our results highlight how different ecoregions in West Africa exhibit different patterns in the benefits people can obtain from trees.

Another simple, yet far-reaching result, is that increases in tree cover above 30% will not necessarily increase children's consumption of vitamin A–rich foods. While we acknowledge that higher tree cover is critical for biodiversity, our results challenge the assumption of a linear relationship between tree cover and people's dietary diversity (39). This result is similar to the findings from Ickowitz et al. (9) who showed how consumption of dark green leaves peaks at relatively low levels of tree cover before declining. Our results also support previous assessments showing that areas outside forests, such as savanna areas, may provide important ecosystem services likely to be beneficial to diets (13). Yet, savannas are often neglected in restoration efforts as they are often seen as degraded forests (28, 44). The finding that high tree cover might do little to secure the consumption of vitamin A–rich foods can be due to a number of possible explanations. First, forest-based pollinators increase the production of domestic fruits in nearby areas, meaning that many scattered trees around the landscape might lead to more effective pollination than bigger areas of forest (45). Second, households are more prone to collect wild fruits and vegetables when traversing landscapes with many scattered trees rather than going deep into interior forest (40). This has, for example, been observed in Nigeria, where a recent study showed how vitamin A–rich foods (e.g. bush mango [Irvingiaceae]) were sourced more frequently among forest-edge communities when compared with interior forest communities (18). Third, in landscapes with sparse tree cover, there might be smaller areas of “managed forests” where valuable fruit trees are consciously maintained (46). Fourth, landscapes with very sparse tree cover could fail to supply diverse vitamin A–rich foods, resulting in people purchasing more of these foods. Finally, landscapes with high tree cover might include forests that are managed for conservation with restricted access for communities (47). Future work should seek to create more explicit evidence on which of these explanations are prevalent in the Sahel-Sudan and lowland forest-savanna ecoregions.

Moreover, our analysis sheds light on the effects that trees can have on dietary quality across poverty levels. In the Sahel-Sudan, we show that increased tree cover has a positive effect across all poverty levels. This suggests that the absence of access to (free) vitamin A–rich foods from trees might not always be compensated by purchasing such foods instead. It also underlines the importance of trees for the intake of vitamin A for a large population in the Sahel-Sudan. Nevertheless, we observe that although higher tree cover in general is beneficial, the least poor households appear to benefit marginally more from greater tree cover than the poorest. Tree-planting and regrowth initiatives, such as the Great Green Wall, should therefore ensure that additional trees would benefit all layers of society (42). Such focus is timely given the shift in species composition recently observed in the Sahel-Sudan; shrubs and highly drought-tolerant and exotic tree species are increasing, whereas traditionally used, multifunctional species and larger trees are decreasing (48). In the lowland forest-savanna ecoregion, we see that the poorest people benefit the most from relatively low levels of tree cover, while the least poor benefit from higher levels of tree cover. In contrast to the Sahel-Sudan, low levels of tree cover in the forest-savanna ecoregion might still host dark green leaves, which are an important and easily accessible source of vitamin A, especially for the poor (Fig. S2). Any tree-planting and regrowth initiatives in the lowland forest-savanna, especially in deforested areas, would have to ensure that benefits from additional tree cover also spread to the poorest people.

In summary, our analysis links data on trees inside and outside of forests to people's food consumption and poverty levels on a broad scale. We show how the presence of trees can potentially be important for the intake of vitamin A–rich foods—but that the effect is not linear as it levels off with more tree cover in the landscape. The next research step could be to examine how the presence of trees influences agricultural diversity, yields, and crop nutritional content (49). New remote sensing techniques that identify specific tree species would allow us to untangle the effects of trees and identify trees with the highest contribution to dietary quality (50). Moreover, studies linking trees outside of forests to people's diets would benefit from more detailed food consumption data recording the quantity of different foods consumed. Such data would allow for a better understanding of how trees affect people's micronutrient intake—not just their intake of specific foods. This is particularly important in West Africa, where micronutrient deficiencies are widespread, with 57% of the population being moderate or severe food insecure and 86% of the population unable to afford a healthy diet (2). Lastly, future studies would benefit from gathering data on where households source their food from, to illuminate whether (and to what extent) people are (i) consuming nutritious foods directly from trees scattered in the landscape or more dense forests or (ii) purchasing more nutritious foods.

## Methods

### Data

We used DHS household data from 10 countries in western Africa (Table S3). The DHS provides information on a range of socioeconomic and health variables and is available at https://dhsprogram.com/data/available-datasets.cfm. In total, this study uses DHS data from 15,875 households from 2,587 rural clusters with at least one child aged 12–59 months. Clusters roughly correspond to a village. DHS data were collected between 2014 and 2019 across the 10 countries in western Africa. Geolocations are available for all clusters, but are displaced for confidentiality purposes. The displacement in a random direction is up to 5 km for 99% of the clusters, and up to 10 km for the remaining 1% of the clusters. To account for the displacement in geolocations, we used a 5-km buffer around each geolocation when spatially overlaying the household data with tree cover data and other spatial data.

### Consumption of vitamin A–rich foods

The DHS interviewed women in the households about their children's food consumption over the previous 24 h. Women were asked whether their children had consumed any foods from 18 predetermined food groups (e.g. eggs and dairy). We chose to focus on the consumption of vitamin A–rich foods by children aged 12–59 months in this study due to (i) forests (and trees outside of forests) anticipated to host a multitude of vitamin A–rich foods, such as wild mango, and (ii) the high rates of vitamin A deficiency in many parts of Africa, which makes it paramount to identify avenues to improve vitamin A intake (24). Thus, we combined the three DHS food groups—vitamin A–rich fruits, vitamin A–rich vegetables and tubers—and dark green leafy vegetables to assess children's overall intake of vitamin A–rich foods. For the analysis presented in Fig. S2, we only included the consumption of dark green leafy vegetables.

### Tree cover

We used tree cover data from a recent continental map of African tree cover in 2019 (12). Based on a deep-learning model applied to 3 m PlanetScope imagery, this dataset includes tree cover from both forest and non-forest trees. For our analysis at the household level, the binary tree cover data were first aggregated to percent cover at 100 m resolution. The tree cover variable was then calculated as the mean percent tree cover in a 5-km buffer around each household.

### Living standards

We were specifically interested in whether the effect of tree cover on the consumption of vitamin A–rich foods would vary across different levels of poverty. We used the living standards dimension of the MPI (MPI-LS (25)), as an indicator of the asset poverty of each household (Table S4). The living standards dimension varies from 0 to 1, with 1 being the most deprived. The MPI-LS is composed of six indicators: (i) assets, (ii) electricity, (iii) sanitation, (iv) cooking fuel, (v) water source, and (vi) housing. Each indicator has an equal weight.

### Ecoregion

We only included ecoregion as a covariate in the model that included an interaction with ecoregion (presented in Fig. 2) and did not differentiate between ecoregions in our main analysis (presented in Fig. 1). Our study area covers four major ecoregions (27), which we lumped into two main ecoregions that represent the ecosystems in the north and south of our study area. Our first region consists of the Sahelian acacia savanna and Sudanian savanna (west and east) ecoregions. Our second region consists of the forest-savanna (Guinean and north-Congolian) and lowland forest (western Guinean, eastern Guinean, and Nigerian) ecoregions. We removed 319 clusters that overlapped ecoregions that are very small (e.g. deserts and xeric shrublands, montane forests, flooded savannas, and mangroves). We calculated the overlap of each cluster (and thus household) with each main ecoregion and assigned each household to the ecoregion with the largest overlap. To increase the contrast in this analysis, we removed 127 clusters that overlapped our two main ecoregions.

### Additional covariates

We controlled for a suite of biophysical and socioeconomic covariates known to influence people's diets—and thus potentially their consumption of vitamin A–rich foods. First, we controlled for household characteristics that are important predictors of household food consumption: household size (31), age of the household head (51), number of children under 5 years old, and the education dimension of the MPI (52) (Table S4). Second, we controlled for agricultural characteristics (53): from the DHS, we extracted tropical livestock units (54) and agricultural land cover from Zanaga et al. (55). Third, we controlled for whether a conflict occurred in the past year before the survey, as (i) conflicts have occurred in many West African countries over the last decade (56), and (ii) conflicts are known to influence people's food intake (57). Fourth, we included geographical covariates that influence land suitability and access (58): slope, percentage of area covered by water, population density, and travel time to the nearest densely populated area. Geographical variables were calculated as the mean value within 5 km of the geolocated household. Finally, we included the country and season (dry/wet) during which the survey was conducted as dummy variables. Additional descriptions and data sources for all covariates are available in Table S5.

### Analysis

We used matching and nonlinear regression techniques to assess the effects of tree cover on the consumption of vitamin A–rich foods and dark green leafy vegetables. We conducted all statistical analyses in R (59).

We used CBGPS (36) to generate weights that adjust for the nonrandom distribution of tree cover, our predictor variable of interest. CBGPS is a form of propensity score matching and, in contrast to most other forms of matching, can be used when the predictor variable of interest is continuous. The CBGPS method produces weights that minimize the correlation between the treatment (tree cover) and covariates (Fig. S3). These weights are then used in subsequent models to reduce endogeneity between tree cover and other covariates, thereby reducing potential bias in the estimates of tree cover on consumption of vitamin A–rich foods.

After matching, we used GAMs to analyze the effect of tree cover on the dependent variable. GAMs have the advantage that they allow for nonlinear relationships. Linear models use a single coefficient to model the relationship between predictor and outcome variables, but GAMs use a smooth function that allows the relationship to vary along the gradient of the predictor. Therefore, by using GAMs, we can assess the effect of tree cover across the entire gradient from bare ground to forest at each percentage of tree cover. We used the *mgcv* package in R to fit GAMs (37). In our main model setup, we included parametric terms for country, education, season, and conflict. We then included numeric covariates as cubic regression smoothing splines, because they take into account both smoothness and local influence, and are widely used in models for nonlinear data (60). To reduce spatial autocorrelation, we included geographic coordinates (latitude and longitude) as a (default) thin smoothing spline.

Our main model takes the form:


$$g(\mu_{i})=X_{i}\beta +f_{m}(T_{m})+f_{1}(x_{1})+\cdots +f_{m}(x_{m})+f(\mathrm{lat},\mathrm{long}),$$

where *g*(*μ_i_*) represents the response variable, *X_i_β* is the parametric part of the linear predictor, *f*_m_(*T*_m_) represents the modeled smooth of tree cover *T*_m_, *f*_m_(*x*_m_) are modeled smooths of numeric covariates, and *f*(lat, long) is the modeled smooth of geographic location.

We used the bam function for large datasets, the default fREML method, the argument discrete = TRUE to ensure model convergence, and a quasi-binomial distribution to model the consumption of vitamin A–rich foods.

Because GAMs do not assume linear relationships, they are also better at capturing complicated interactions between predictor variables than linear models. We assessed the interaction between tree cover and living standards by adding an interaction effect. We did so by adding a tensor product smooth (“ti” function) to the main model setup. To assess the interaction effect separately for each ecoregion, we also included ecoregion as a parametric term and allowed the interaction (i.e. tensor product smooth) to vary for each ecoregion. Our model thus takes the form:


$$\begin{array}{rl} g(\mu_{i}) & =X_{i}\beta +f_{m}(T_{m})+f_{1}(x_{1})+\cdots +f_{m}(x_{m})+f_{\mathrm{geo}}(\mathrm{lat},\mathrm{long})\\ & \;+f_{m}(T_{m},\upsilon_{m})Z_{m}, \end{array}\;$$

where *f*_m_(*T*_m_, *υ*_m_) represents the interaction between tree cover *T*_m_ and living standards *υ*_m_, and *Z*_m_ is the ecoregion.

Finally, we used the DHARMa package (61) to calculate Moran's *I* as an estimate of spatial autocorrelation. Although spatial autocorrelation was still present in the residuals after including a smooth of the geographic location, Moran's *I* was very low (0.01). We thus conclude that there is no evidence that spatial autocorrelation influences our estimates.

## Supplementary Material

pgae067_Supplementary_Data

## Data Availability

All data needed to replicate our results are available online. Our analysis uses publicly available data from the Demographic Health Survey (DHS), available at https://dhsprogram.com/data/available-datasets.cfm. Maps on tree cover are available at https://doi.org/10.5281/zenodo.7764460. Ecoregion data are available from https://ecoregions.appspot.com/. Geographic data on covariates were downloaded using Google Earth Engine (see Table S5 for references). Code is available from B.d.B. (bowydenbraber@gmail.com).
