# Supplementary material for: Even low levels of tree cover improve dietary quality in West Africa
Source: PNAS Nexus. 2024 Feb 9;3(2):pgae067. doi: 10.1093/pnasnexus/pgae067 (PMC10890828; doi:10.1093/pnasnexus/pgae067)
Supplement: pgae067_Supplementary_Data [file pgae067_supplementary_data.docx]

**Supplementary Information**

**Table S1. Summary statistics for all variables across ecoregions.** All values are means with standard deviations in parentheses, with the exception of vitamin A consumption (of fruits and vegetables), where we report the proportion of households consuming the food group. N=15,875 households from 2,587 rural clusters.

| **Variable** | **Sahel-Sudan**      **Mean**  **(SD)** | **Lowland forest-Savanna**    **Mean**  **(SD)** |
| --- | --- | --- |
| Tree cover (%) | 7.925  (0.091) | 40.686  (0.342) |
| Vitamin A consumption | 0.406  (0.005) | 0.498  (0.006) |
| Living Standards (MPI-LS) | 0.660  (0.002) | 0.642  (0.003) |
| Education (MPI-Ed) | 0.646  (0.004) | 0.438  (0.005) |
| Agricultural land cover (%) | 0.435  (0.003) | 0.130  (0.003) |
| Occurrence of water (%) | 0.011  (0.000) | 3.023  (0.002) |
| Slope (degrees) | 1.341  (0.011) | 3.606  (0.034) |
| Tropical Livestock Unit (TLU) | 4.135  (0.081) | 1.213  (0.052) |
| Population density in 2000 (Estimated number of persons per km^2^) | 121.210  (3.234) | 174.882  (4.977) |
| Travel time to nearest densely populated area (minutes) | 108.334  (1.157) | 80.429  (1.050) |
| Household size | 9.943  (0.073) | 8.015  (0.061) |
| Age of household head (years) | 42.929  (0.140) | 44.835  (0.193) |
| Age of children (months) | 29.441  (0.108) | 27.871  (0.145) |
| Number of children under 5 years | 2.845  (0.018) | 2.242  (0.018) |

**Figure S1: Spatial distribution of DHS data.** Top) Two ecoregions, covering 10 countries, Middle) Tree cover across DHS clusters. Map features 2019 PlanetScope data with tree cover predictions at 3m resolution, and Bottom) Multidimensional Poverty Index Living Standards (MPI-LS) across DHS clusters. N=15,875 households from 2,587 rural clusters.


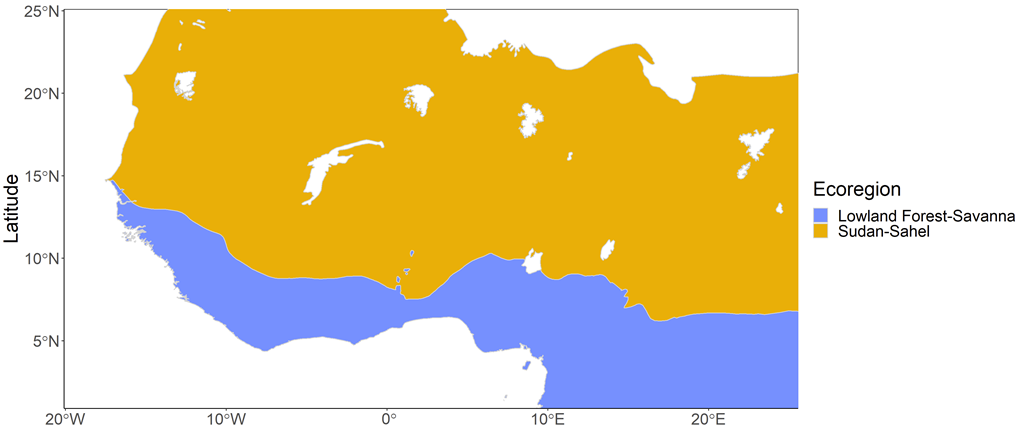


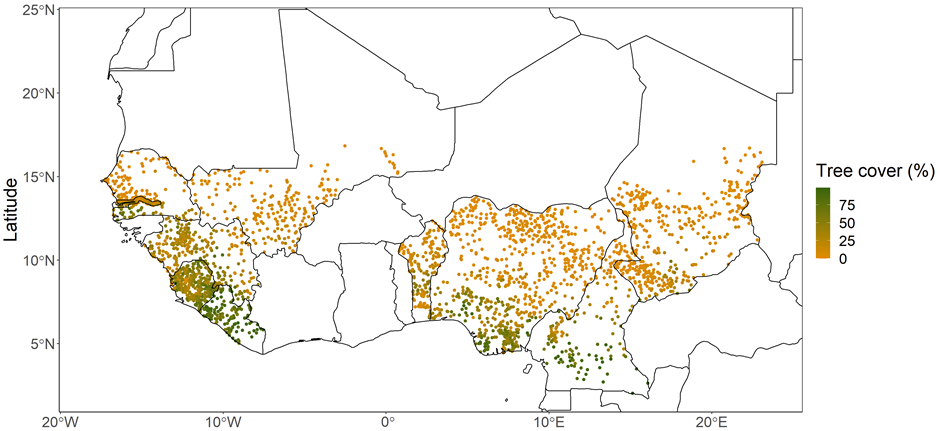

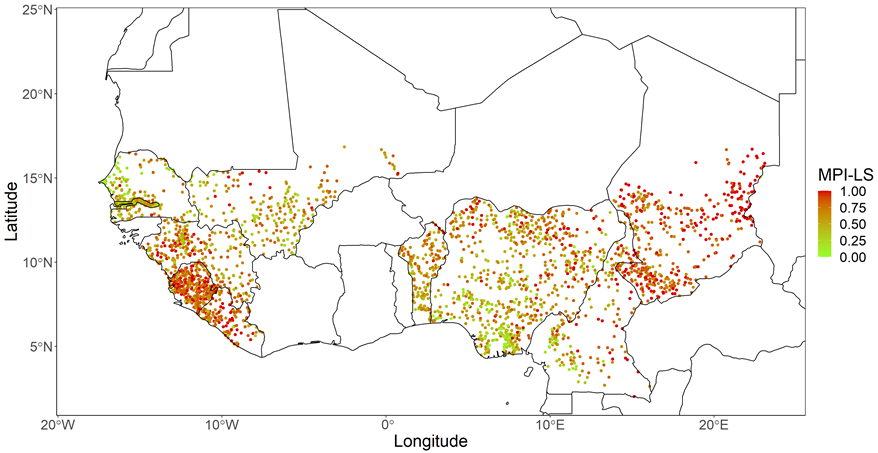


**Figure S2: Impacts of tree cover on consumption of dark green leafy vegetables across all the countries (a), and per ecoregion and poverty level (b).** N=81,296 rural households in Senegal, Mali, Nigeria, Gambia, Benin, Guinea, Chad, Liberia, Sierra Leone, and Cameroon. Lines indicate the fitted probability that the children in a household consumed dark green leafy vegetables - red line indicates high level of poverty (multidimensional poverty index (MPI) > 0.83), blue line indicates low level of poverty (MPI < 0.33). The error shows the credible interval. The points indicate average probability of consumption of groups of households with similar tree cover level and MPI. The top chart shows the frequency of household observations across tree cover values**.**


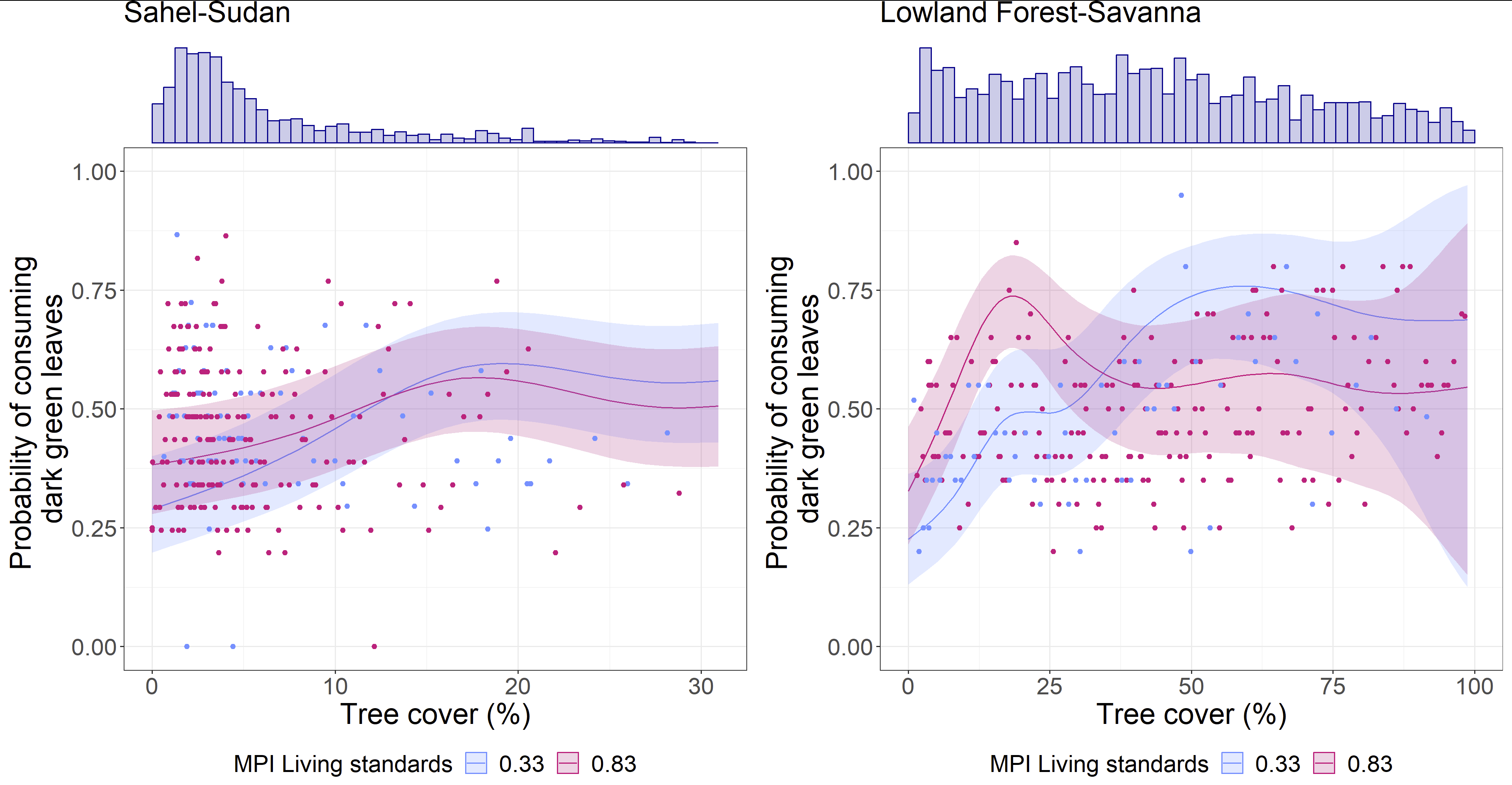

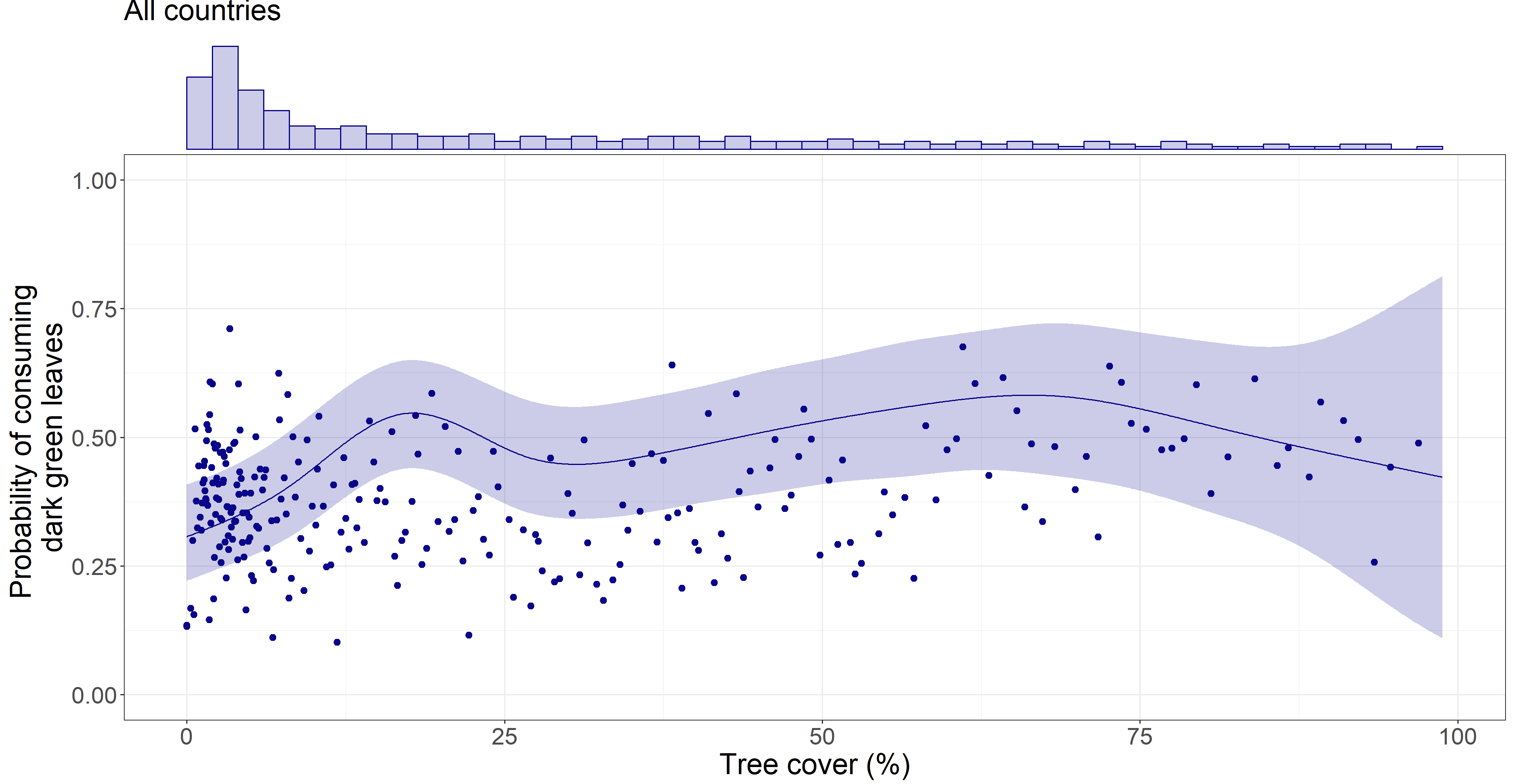


**a**

**b**

**Figure S3: Balance of covariates before and after CBGPS matching.** For all models the ‘treatment’ variable was tree cover in a 5 km radius circle around households. Absolute Pearson correlations for confounding covariates before matching (orange circles) and after matching (green circles).


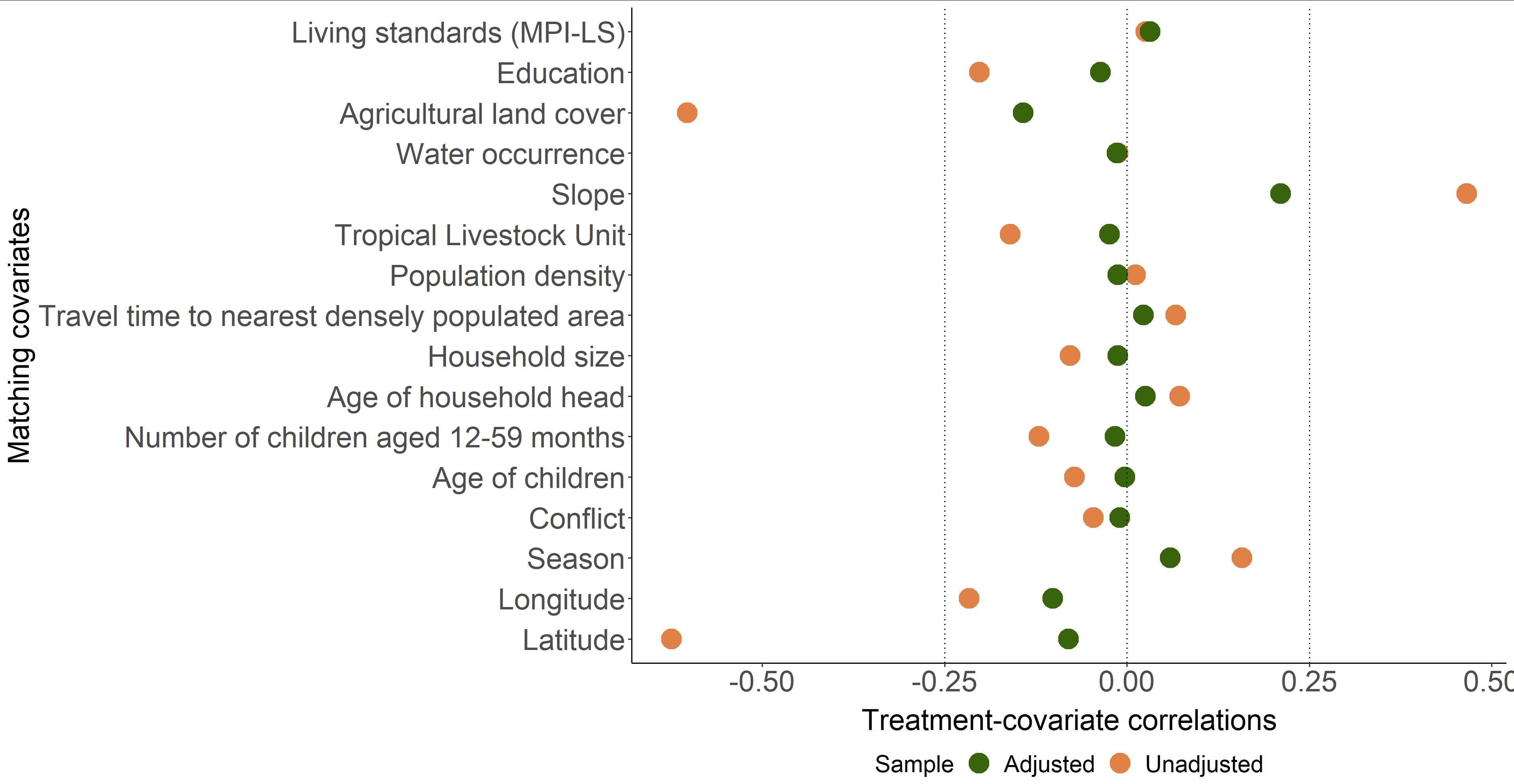


**Table S2: Useful tree species in West Africa for the Sahel-Sudan and lowland forest-savanna ecoregions. Tree species and uses are extracted from** [**https://www.worldagroforestry.org/output/useful-tree-species-africa**](https://www.worldagroforestry.org/output/useful-tree-species-africa)**. The species are selected to provide a few examples rather than an exhaustive list of species present in the two regions.**

| **Ecoregion** | **Major vegetation type** | **Example species** | **Common name** | **Uses** |
| --- | --- | --- | --- | --- |
| **Sahel-Sudan** | Desert  Semi-desert vegetation  Woodland  Forest transitions and mosaics | Boscia senegalensis  Maerua crassifolia  Salvadora persica  Acacia macrostachya  Annona senegalensis  Balanites aegyptiaca  Commiphora pedunculata  Canarium schweinfurthii  Ricinodendron heudelotii  Antidesma venosum | Hanza  Toothbrush tree  Wild custard apple  Egyptian balsam  Bush candle  Cocoa’s friend Tassel berry | Edible fruits and seeds  Edible leaves  Edible fruits and leaves  Edible seeds  Edible fruits  Edible fruits, seeds, leaves  Edible fruits  Edible fruits  Edible fruits and leaves  Edible fruits |
| **Lowland Forest Savanna** | Forest  Woodland | Canarium schweinfurthii  Ricinodendron heudelotii  Carapa procera  Acacia macrostachya  Annona senegalensis  Balanites aegyptiaca  Commiphora pedunculata | Bush candle  Cocoa’s friend  African crabwood  Wild custard apple  Egyptian balsam | Edible fruits  Edible fruits and leaves  Nuts collected for oil  Edible seeds  Edible fruits  Edible fruits, seeds, leaves  Edible fruits |

**Table S3: Countries with DHS data, year of survey, associated number of household observations used to assess effects of tree cover on consumption of vitamin A-rich foods, and mean values for tree cover and MPI Living Standards**

| **Country** | **Year of survey** | **Number of households with children aged 12-59 months** | **Tree cover (%)** | **Proportion of children who consumed Vitamin A-rich foods** | **Living Standards (MPI)** |
| --- | --- | --- | --- | --- | --- |
| Benin | 2017-18 | 999 | 16.8 | 0.403 | 0.659 |
| Cameroon | 2018 | 1,320 | 39.0 | 0.541 | 0.679 |
| Chad | 2014-15 | 2,355 | 6.7 | 0.272 | 0.881 |
| Gambia | 2019-20 | 1,811 | 17.4 | 0.232 | 0.515 |
| Guinea | 2018 | 833 | 31.4 | 0.513 | 0.653 |
| Liberia | 2019-20 | 525 | 79.3 | 0.590 | 0.785 |
| Mali | 2018 | 1,254 | 6.7 | 0.575 | 0.597 |
| Nigeria | 2018 | 4,332 | 19.8 | 0.487 | 0.601 |
| Senegal | 2019 | 1,091 | 15.1 | 0.501 | 0.468 |
| Sierra Leone | 2019 | 1,355 | 45.5 | 0.570 | 0.797 |

**Table S4: Description of components included in the multidimensional poverty index (MPI)**

| **Dimension** | **Indicator** | **Criteria** |
| --- | --- | --- |
| Living standards | Electricity | Household has no electricity |
|  | Sanitation | Household has no improved sanitation or improved but shared with another household based on MDG guidelines |
|  | Drinking water | Household does not have access to safe drinking water, or the drinking water source is more than 30 minute walk roundtrip (Based on MDG guidelines) |
|  | Floor material | Household has a sand, dirt or mud floor |
|  | Clean Cooking fuel | Household uses firewood, charcoal as cooking fuel |
|  | Assets | Household does not have more than two of the following assets (radio, television, refrigerator, bicycle, motorcycle, telephone, computer, animal cart) and does not a own a car or a truck |
| Education | Years of schooling | Any household member does not have six years of education aged 13 – years and older |
|  | Child school attendance | Any Child of school aged 6 – 12 years does not attend school |

**Table S5: Description of covariates that potentially influence people’s diets**

| **Variable** | **Spatial resolution** | **Year** | **Reference** | **Notes** |
| --- | --- | --- | --- | --- |
| Population in 2000 (Estimated number of persons per km^2^) | 1,000 meter | 2000 | CIESIN (51) |  |
| Travel time to nearest densely populated area (minutes) | 1,000 meter | 2015 |  | Travel time calculations were performed in Google Earth Engine, based on the friction map of Weiss *et al.* (52) |
| Occurrence of permanent water bodies (%) | 30 meter | 2000 | Pekel *et al.* (53) |  |
| Slope (degree) | 90 meter | 2000 (SRTM source data) | SRTM (54) |  |
| Agricultural land cover | 10 meter | 2021 | ESA WorldCover 2021 v200 Zanaga et al. (55) |  |
| Tropical livestock unit (TLU) |  | Year of survey | DHS | TLU calculations were based on ref (56) |
| Conflict (binary) |  | Up to one year before the survey | Uppsala Conflict Data Program (57) | Conflicts aggravate food security of households and affect consumption of multiple food groups (58). |
| MPI – Education |  | Year of survey | DHS |  |
| Household size |  | Year of survey | DHS |  |
| Age of household head |  | Year of survey | DHS |  |
| Average age of children in household |  | Year of survey | DHS |  |
| Number of children under 5 years |  | Year of survey | DHS |  |

**References for supplementary information:**

1. [dataset] UN WPP-Adjusted Population Count, v4.11: Gridded Population of the World (GPW), v4 | SEDAC (April 19, 2023).

2. [dataset] D. J. Weiss, *et al.*, A global map of travel time to cities to assess inequalities in accessibility in 2015. *Nature* **553**, 333–336 (2018).

3. [dataset] J.-F. Pekel, A. Cottam, N. Gorelick, A. S. Belward, High-resolution mapping of global surface water and its long-term changes. *Nature* **540**, 418–422 (2016).

4. [dataset] SRTM 90m Digital Elevation Database v4.1. *CGIAR-CSI* (2017) (April 19, 2023).

5. [dataset] D. Zanaga, *et al.*, ESA WorldCover 10 m 2021 v200 (2022) https:/doi.org/10.5281/zenodo.7254221 (July 14, 2023).

6. FAO, *Guidelines on methods for estimating livestock production and productivity* (FAO, 2018).

7. [dataset] N. P. Gleditsch, P. Wallensteen, M. Eriksson, M. Sollenberg, H. Strand, Armed Conflict 1946-2001: A New Dataset. *Journal of Peace Research* **39**, 615–637 (2002).

8. C. P. Martin-Shields, W. Stojetz, Food security and conflict: Empirical challenges and future opportunities for research and policy making on food security and conflict. *World Development* **119**, 150–164 (2019).
